# Plasma Branched-Chain Amino Acids and Risk of Incident Type 2 Diabetes: Results from the PREVEND Prospective Cohort Study

**DOI:** 10.3390/jcm7120513

**Published:** 2018-12-04

**Authors:** Jose L. Flores-Guerrero, Maryse C. J. Osté, Lyanne M. Kieneker, Eke G. Gruppen, Justyna Wolak-Dinsmore, James D. Otvos, Margery A. Connelly, Stephan J. L. Bakker, Robin P. F. Dullaart

**Affiliations:** 1 Department of Internal Medicine, UMCG, University of Groningen, 9713 GZ Groningen, The Netherlands; m.c.j.oste@umcg.nl (M.C.J.O.); l.m.kieneker@umcg.nl (L.M.K.); s.j.l.bakker@umcg.nl (S.J.L.B.); 2 Department of Endocrinology, UMCG, University of Groningen, 9713 GZ Groningen, The Netherlands; e.g.gruppen@umcg.nl (E.G.G.); r.p.f.dullaart@umcg.nl (R.P.F.D.); 3 Laboratory Corporation of America Holdings (LabCorp), Morrisville, NC 27560, USA; wolakdj@labcorp.com (J.W.-D.); otvosj@labcorp.com (J.D.O.); connem5@labcorp.com (M.A.C.)

**Keywords:** branched-chain amino acids, risk factor, type 2 diabetes, insulin resistance

## Abstract

Plasma branched-chain amino acids (BCAAs) are linked to metabolic disease, but their relevance for prediction of type 2 diabetes development is unclear. We determined the association of plasma BCAAs with type 2 diabetes risk in the prevention of renal and vascular end-stage disease (PREVEND) cohort. The BCAAs were measured by means of nuclear magnetic resonance spectroscopy. We evaluated the prospective associations of BCAAs with type 2 diabetes in 6244 subjects. The BCAAs were positively associated with HOMA-IR after multivariable adjustment (*p* < 0.0001). During median follow-up for 7.5 years, 301 cases of type 2 diabetes were ascertained. The Kaplan-Meier plot demonstrated that patients in the highest BCAA quartile presented a higher risk (*p* log-rank < 0.001). Cox regression analyses revealed a positive association between BCAA and type 2 diabetes; the hazard ratio (HR) for the highest quartile was 6.15 (95% CI: 4.08, 9.24, *p* < 0.0001). After adjustment for multiple clinical and laboratory variables, the association remained (HR 2.80 (95% CI: 1.72, 4.53), *p* < 0.0001). C-statistics, Net reclassification improvement, and −2 log likelihood were better after adding BCAAs to the traditional risk model (*p* = 0.01 to <0.001). In conclusions, high concentrations of BCAAs associate with insulin resistance and with increased risk of type 2 diabetes. This association is independent of multiple risk factors, HOMA-IR and β cell function.

## 1. Introduction

Amino acids have an important function in addition to building proteins; they are also critical intermediaries of intracellular signaling [1]. Branched-chain amino acids (BCAAs) are amino acids that have non-linear aliphatic side-chains, and include the essential amino acids leucine, valine, and isoleucine. Most of the essential amino acids are metabolized in the liver, whereas BCAAs are catabolized under the joint control of both skeletal muscle and the liver [2]. 

Over the last years, the association of BCAAs with obesity, insulin resistance, and diabetes risk has received more attention, and is reflected in the increased number of publications. From experimental studies in murine models to clinical reports based on food frequency questionnaires and metabolomics approach, the evidence points to BCAAs as a relevant factor in the pathogenesis of dysglycemia and the metabolic syndrome [3].

Oxidation of BCAAs in muscle has been linked to glucose homeostasis, but there is equivocal evidence regarding the role of BCAAs on insulin sensitivity. Some studies suggest that BCAAs may improve muscle glucose uptake by enhancing glucose recycling via the glucose–alanine cycle and that they may contribute to the regulation of insulin signaling [4]. However, other studies in humans and in animal models have reported that increased plasma concentrations of leucine have no effect [5] or may even increase insulin resistance via the inhibitory serine phosphorylation of insulin receptor substrate-1 [6]. In addition, it has been demonstrated that leucine deprivation increases hepatic insulin sensitivity [7]. 

The biochemical mechanism underlying the association of BCAAs with insulin resistance has been approached in several studies. Newgard et al. [8] have reported that murine models fed with BCAAs and a high-fat diet presented accumulation of mitochondrial acylcarnitines, which lead to insulin resistance. They demonstrated that BCAAs plays a particular role in the chronic activation of the mammalian target of rapamycin (mTOR) protein kinase, which was not explained only by the high-fat diet. 

There is also epidemiological evidence to suggest a positive cross-sectional association of circulating concentrations of BCAAs with insulin resistance, and it had been suggested that BCAAs are relevant for type 2 diabetes development [9,10]. In line, using data from two independent cohorts, we have recently reported that high circulating concentrations of BCAAs are associated with the presence of type 2 diabetes and metabolic syndrome [11].

Some studies have also explored the prospective association of BCAAs with glycemia [12] and incidence of type 2 diabetes. Wang et al. [13] reported the association of individual BCAAs with type 2 diabetes incidence in two nested case-control studies with 704 participants in total. In another study with 526 participants being followed for 4.7 years, the association of BCAAs and incident type 2 diabetes did not remain significant after adjustment for insulin resistance [14]. A positive association of circulating BCAAs with incident type 2 diabetes has also been reported for an Asian population [15]. Recently, Ruiz-Canela et al. [16] reported a positive association between plasma BCAAs with type 2 diabetes incidence in a case-cohort study among European subjects followed for 3.8 years. However, given the fact that in a case-cohort study cases are overrepresented, there are limitations in the assessment of prediction measures in such studies [17,18]. Previous studies have limitations in terms of sample size, design and follow-up. For that reason, it is unclear whether circulating concentrations of BCAAs have the ability of actually improving prediction of an established type 2 diabetes risk model. We, therefore, determined the extent to which BCAA plasma concentrations, i.e., the sum of valine, leucine, and isoleucine, can improve risk prediction of type 2 diabetes incidence, in the prevention of renal and vascular end-stage disease (PREVEND) in a prospective population-based cohort study.

## 2. Materials and Methods

### 2.1. Study Population

The PREVEND study was a prospective population-based cohort study in Groningen, the Netherlands. The design of the PREVEND study has been described in detail elsewhere [19,20]. Briefly, from 1997 to 1998, all residents from Groningen aged 28–75 years were invited to participate. Pregnant women, type 1 diabetic subjects, and type 2 diabetic subjects using insulin were not allowed to participate. All participants with a urinary albumin concentration ≥10 mg/L were invited to our clinic together with randomly selected subjects with a urinary albumin concentration <10 mg/L, and 8592 individuals completed an extensive examination. 

For the present analysis, we conducted a post-hoc analysis using data from participants who completed the second screening round, excluding those with missing values of BCAA concentrations (*n* = 1901) or pre-existing type 2 diabetes (*n* = 447), leaving a cohort of 6244 participants with complete information for analysis. The protocol for the PREVEND study was approved by the local ethics committee of the University Medical Center Groningen. All participants in the present analysis provided written informed consent to participate and all study procedures were conducted according to the Declaration of Helsinki.

### 2.2. Baseline Assessment of BCAA

During two outpatient visits, baseline data were collected on demographics, lifestyle factors, anthropometric measurements, medical history, as well as prevalent medical conditions and use of medication. Plasma samples were taken from participants after an overnight fast and 15 min of rest prior to sample collection. All blood samples were taken between 8:00 and 10:00. Plasma samples were prepared by centrifugation at 4 °C and were stored at −80 °C until analysis. 

Plasma valine, leucine, and isoleucine concentrations were measured using a Vantera Clinical Analyzer (LabCorp, Morrisville, NC, USA)—a fully automated, high-throughput, 400 MHz proton (1H) nuclear magnetic resonance (NMR) spectroscopy platform. Plasma samples were prepared on board the instrument, and automatically delivered to the flow probe in the NMR spectrometer’s magnetic field. The validation of the use of NMR for quantification of BCAAs has previously been described by our group [10,11]. Data acquisition on the Vantera and the spectra data processing have been reported in greater detail elsewhere [21]. 

### 2.3. Clinical and Laboratory Measures

Height and weight were measured with the participants standing without shoes and heavy outer garments. Body mass index (BMI) was calculated by dividing weight in kilograms by height in meters squared. Systolic and diastolic blood pressure values were recorded as the means of the last two recordings of the second visit. Total cholesterol, triglycerides, insulin, serum creatinine, and serum cystatin C were measured using standard protocols, which have been previously described [22,23,24,25]. Urinary albumin excretion (UAE) was measured as described in two 24-h urine collections and the results were averaged for analysis [23,24,25]. Fasting plasma glucose was measured by dry chemistry (Eastman Kodak, Rochester, NY, USA). The homeostatic model assessment for insulin resistance (HOMA-IR) was calculated as fasting plasma insulin (mU/L) × fasting plasma glucose (mmol/L)/22.5. The homeostatic model assessment for beta cell function (HOMA-β) was calculated using the equation: 20 × fasting plasma insulin (mU/L)/(fasting plasma glucose (mmol/L) − 3.5). The homeostatic model assessment for beta cell represents the relative β-cell function of an individual and is expressed as a percentage. Estimated glomerular filtration rate (eGFR) was calculated using the Chronic Kidney Disease Epidemiology Collaboration (CKD-EPI) combined creatinine–cystatin C equation [26].

### 2.4. End Point of the Study

Participants were followed from the date of the baseline center visit until end of follow-up. Incident type 2 diabetes was established if one or more of the four criteria were met during follow-up: (1) blood glucose ≥ 7.0 mmol/L (126 mg/dL); (2) random sample plasma glucose ≥ 11.1 mmol/L (200 mg/dL); (3) self-report of a physician diagnosis; (4) initiation of glucose lowering medication according to the central pharmacy registry follow-up data, which was completed as of 1 January 2011.

### 2.5. Statistical Analysis 

Variables with a nonlinear distribution were natural log transformed. Data were presented as the mean (standard deviation, SD) or median (interquartile range, IQR) for continuous variables and percentages for categorical variables. Cross-sectional associations at baseline were assessed by multivariable linear regression for continuous variables and by χ^2^ test for categorical variables. Results of cross-sectional associations of BCAAs with insulin resistance and pancreatic β-cell function are presented as unstandardized regression coefficients and 95% confidence intervals (CIs).

For the prospective analysis, we plotted cumulative Kaplan-Meier curves for type 2 diabetes development during follow-up according to quartiles of BCAAs. Time-to-event Cox proportional hazards models were used to assess the hazard ratio (HR) and 95% CI of incident type 2 diabetes among 6244 participants free of type 2 diabetes at baseline. Hazard ratios were calculated in 5 adjusted models: for (1) age and sex; (2) plus family history of type 2 diabetes and BMI; (3) plus alcohol consumption and smoking status; (4) plus triglycerides; (5a) plus HOMA-IR; (5b) and HOMA-β; (5c) plus HOMA-IR and HOMA-β. Possible effect modification was explored by including the interaction terms between BCAAs and age or sex in the multivariable adjusted models. These analyses were conducted using valine, leucine, and isoleucine separately and its sum (BCAA) as independent variables.

In order to determine whether BCAA values can improve the predictive ability of a conventional model [27], we calculated measures of discrimination for censored time-to-event data (Harrell’s C-index) [28] and reclassification. In order to evaluate the change in C-index in addition to BCAAs, two type 2 diabetes risk prediction models were fitted: first, a model using clinical and laboratory variables (age, sex, family history of type 2 diabetes, BMI, insulin, triglycerides, and fasting plasma glucose), as used by Wilson et al. [29] in the Framingham Offspring Study; and second, a model with the variables mentioned above plus BCAAs. Subsequently, we tested the ability of the combined model with BCAA concentrations to correctly reclassify participants into categories of predicted type 2 diabetes risk. Using predefined risk categories of type 2 diabetes development (<10%), intermediate (10% to 20%), and high (≥20%) [27], reclassification was assessed using the categorical net reclassification improvement (NRI) approach [30]. 

Considering that Harrell’s C-index may not be able to detect differences in risk prediction of potential biomarkers because its calculation is not based on continuous data, but ranks [31], we decided to use the −2 log likelihood test as another sensitive risk discrimination method [32]. For that reason, in addition to Harrell’s C-index, we tested differences in the −2 log likelihood of prediction models with and without inclusion of BCAA values. All statistical analyses were conducted in R version 3.4.2 (Boston, MA, USA). Two-sided *p*-values < 0.05 were considered significant.

## 3. Results

### 3.1. Baseline Characteristics

Baseline characteristics of the 6244 subjects included in the current study are shown in Table 1 (in sex-stratified quartiles). Among them, 50.6% were women and 14.2% reported to have positive family history of type 2 diabetes. Mean BCAA concentration for all participants was 370.3 ± 88.6 μM, valine was 203.08 ± 46.5 μM, leucine was 124.9 ± 32.5 μM, and isoleucine was 42.9 ± 16.1 μM (Table 1). In men, the mean BCAA concentration was 405.40 ± 90.00 μM, which was 366.11 ± 72.43 μM in women (*p* < 0.001). Subjects with the highest quartile of BCAA concentrations were more likely to be older, have higher BMI, blood pressure, and used tobacco more frequently. Additionally, those subjects also presented higher concentrations of total cholesterol, triglycerides, glucose, insulin, HOMA-IR, HOMA-β, and creatinine. The percentages of a positive family history of chronic kidney disease (CKD) and alcohol consumption, as well as the urinary albumin excretion rate were similar among the different quartiles of BCAA (Table 1).

### 3.2. Associations at Baseline 

Branched-chain amino acids were associated with HOMA-IR and HOMA-β in crude as well as in age- and sex-adjusted analyses (Table 2). The positive association of BCAA with HOMA-IR remained after additional adjustment for HOMA-β. However, after adjustment for HOMA-IR, there was an inverse relationship between BCAA with HOMA-β. The associations of BCAA concentrations and other variables of interest were further evaluated with univariable and multivariable regression (Table 3). In univariable analyses, sex, age, race, BMI, systolic and diastolic blood pressure, parental history of type 2 diabetes, alcohol consumption, use of antihypertensive and lipid-lowering drugs, cholesterol, triglycerides, glucose, insulin, creatinine, and UAE were positively associated with BCAA, whereas smoking status, HDL cholesterol, and eGFR were inversely associated. In a multivariable analysis, taking account of all these variables together, the positive associations with BCAA that remained at a *p* < 0.05 were: sex, race, BMI, parental history of type 2 diabetes, alcohol consumption, total cholesterol, triglycerides, and HOMA-IR. High-density lipoprotein cholesterol remained inversely associated (Table 3). Of note, in fully adjusted analysis, BCAA was associated with HOMA-IR but was unrelated to HOMA-β.

### 3.3. Longitudinal Analysis

**Figure 1:**
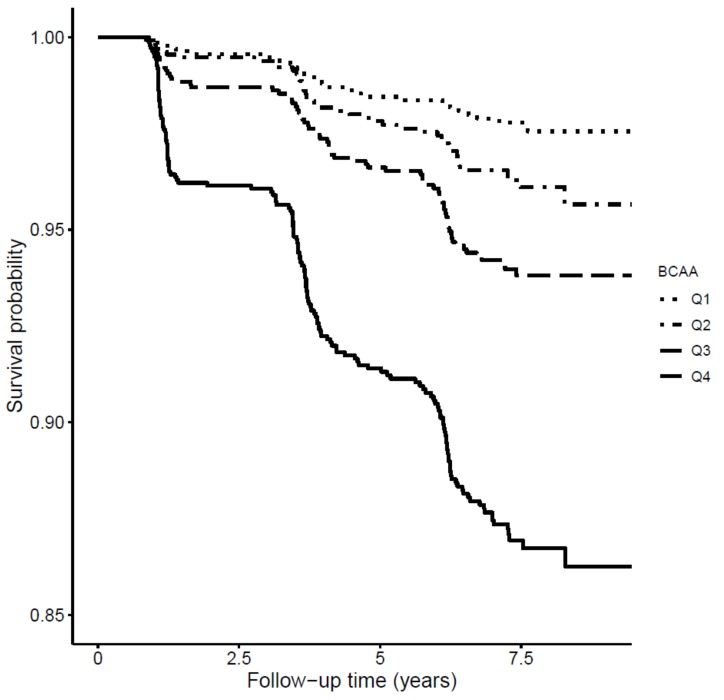
Kaplan-Meier curves for incident type 2 diabetes survival according to quartiles of BCAAs, by log-rank test (*p* < 0.001).

During a median follow-up of 7.5 years (IQR, 7.2–8.0.), 301 participants (4.8%) developed type 2 diabetes (Table 4). The Kaplan-Meier curves for incident type 2 diabetes according to quartiles of BCAA concentrations are presented in Figure 1. The graph revealed an increased risk of type 2 diabetes in the top quartile of BCAA concentrations (*p*-value for log-rank test <0.001). In Cox regression analysis that compared the highest with the lowest quartiles of the distribution of BCAA concentrations adjusted for age and sex; high BCAA concentrations were associated with increased risk of incident type 2 diabetes, showing an HR of 6.15 (95% CI: 4.08, 9.24) (Table 4). The association of type 2 diabetes risk with BCAA remained significant after adjustment for HOMA-IR (HR: 2.80; 95% CI: 1.72, 4.53). Likewise, when BCAA was analyzed as HR per 1 SD increase, the risk of newly developing type 2 diabetes was significant (HR 1.28 (95% CI: 1.13, 1.46)) after adjustment for HOMA-IR (Table 4). There was no statistically significant interaction between BCAA and age or sex on T2D incidence (interactions: *p* = 0.37 and *p* = 0.11, respectively).

Cox regression analyses were also performed with the individual BCAAs in the same models. These analysis essentially showed a similar pattern with diabetes risk. When valine was analyzed as HR per 1 SD increase, the risk of newly developing type 2 diabetes was close to significance (HR 1.13 (95% CI: 0.98, 1.29) per 1 SD increment, *p* = 0.07) in the fully adjusted model (Supplemental Table S1). In the same model, leucine presented a HR similar to BCAA (HR 1.18 (95% CI: 1.03, 1.34) per 1 SD increment, *p* = 0.011) (Supplemental Materials Table S2). Finally, isoleucine showed a significant but marginally weaker association with incident type 2 diabetes (HR 1.11 (95%, CI: 1.00, 1.24) per 1 SD increment, *p* = 0.043) (Supplemental Materials Table S3). 

Stratified analyses were performed for fasting plasma glucose concentrations (using two cut points: 4.7 mmol/L (median) and 5.6 mmol/L (prediabetes cutoff value)). The results of the stratified analyses were essentially similar compared to the main results (data not shown). The HRs adjusted for traditional risk factors in the subset of patients with baseline glucose concentrations <4.7 mmol/L and ≥4.7 mmol/L were 1.47 (95% CI: 1.02, 2.17) and 1.36 (95% CI: 1.20, 1.54), respectively. Using the cutoff values of <5.6 mmol/L and ≥5.6 mmol/L, the HRs were 1.35 (95% CI: 1.10, 1.66) and 1.31 (95% CI: 1.11, 1.54), respectively; (*p*-value <0.001 for all comparisons).

### 3.4. Effect of Inclusion of BCAA on Type 2 Diabetes Risk Prediction

A type 2 diabetes risk prediction model containing established risk factors yielded a C-index of 0.8034 (95% CI: 0.8005, 0.8063). After addition of information on BCAA concentrations, the C-index increased to 0.8057 (95% CI: 0.8028, 0.8086) (*p* < 0.01). The differences of the −2 log likelihood of the type 2 diabetes predictive model with addition of total BCAAs, also showed a significant improvement (*p* = 0.001). The NRI assessment of the participants that remained free of type 2 diabetes revealed that 27% were correctly reclassified to a lower risk category and 10% were reclassified to a higher risk category. There was a significant improvement in the classification of participants into predicted type 2 diabetes risk categories with a NRI of 0.43 (95% CI: 0.31, 0.54) (*p* < 0.0001).

## 4. Discussion

In this large-scale prospective population-based cohort study, we investigated the associations of plasma concentrations of total BCAAs (i.e., valine, leucine, isoleucine) with the risk of type 2 diabetes. Baseline characteristics such as male sex, older age, and high BMI were positively associated with high concentrations of BCAA, coinciding with the findings of other cross-sectional [13] and longitudinal studies [33]. Moreover, BCAA concentrations were positively associated with insulin resistance but not with β cell function in fully adjusted analyses. We found that subjects with high circulating BCAA concentrations presented a significantly higher risk for type 2 diabetes. The association remained significant after adjustment for established risk factors, including age, sex, BMI, parental history of type 2 diabetes, hypertension, alcohol consumption, as well as HOMA-IR and HOMA-β. Addition of BCAA to the traditional predictive model improved its type 2 diabetes predictive ability. Furthermore, the BCAA enriched model improved reclassification of participants across clinical risk categories for type 2 diabetes.

In our study, men had higher BCAA concentrations compared to women. The most recent study about plasma BCAA and type 2 diabetes was conducted in women with a history of gestational diabetes and demonstrated that the positive association of circulating BCAAs and type 2 diabetes is also presented in women [34]. We also found that older participants had the highest values of BCAAs at baseline; however, the association with incident type 2 diabetes was independent of age. Previous studies suggest that the association of BCAAs with metabolic disorders can also be present in young people from 8 to 18 years old [35]. 

Insulin resistance is one of the main factors in the development of type 2 diabetes. In this study it was estimated by HOMA-IR, which fairly correlates with glucose disposal as determined by the hyperinsulinemic euglycemic clamp technique [35]. We found a cross-sectional association of BCAAs with insulin resistance (β = 28.92, *p* < 0.0001) which agrees with our previous smaller studies, that showed insulin resistance to be associated with BCAA, independent of sex, age, type 2 diabetes status, and BMI [11]. Similarly, Shah and colleagues [36] also reported a correlation between BCAA and HOMA-IR. 

Notably, we found no association of BCAAs with HOMA-β in the fully adjusted analysis (Table 2). Previous studies did not evaluate the association of β cell function with BCAAs taking account of insulin resistance [11,12,37]. Other studies only evaluated insulin resistance or fasting plasma insulin [14,36,38,39]. Wang et al. [13] reported the analysis of the potential association of BCAAs with beta cell function, but it was not fully adjusted. Our observation of an independent association of BCAAs with HOMA-IR rather than with HOMA-β reinforces the possible role of insulin resistance as a contributing mechanism in part explaining the association of BCAAs with type 2 diabetes risk. Yet, the main finding of our study is that BCAAs predicted incident type 2 diabetes even when taking into account insulin resistance and β cell function.

The influence of BCAAs on glucose metabolism has been intensively investigated in animal models and humans [9]. Wang-Sattler et al. [38] reported a lack of association of the baseline concentrations of leucine, isoleucine, and valine with glucose tolerance status seven years later in the Cooperative Health Research in the Augsburg Region (KORA) cohort. Nevertheless, we found a significant association between those individual amino acids at baseline and fasting plasma glucose at the last visit of the PREVEND study (*p* < 0.01) (data not shown). 

Earlier studies have shown that circulating concentrations of BCAA are positively associated with incident type 2 diabetes in an Asian population [15]; nonetheless, such association was not significant among the European population of the Southall and Brent REvisited (SABRE) cohort [40]. On the other hand, Ferrannini et al. [37] reported that 130 representative subjects from Bosnia who developed type 2 diabetes after a follow-up of 9.5 years presented increased concentrations of leucine, isoleucine, and valine at baseline, in comparison with the 412 subjects that remained free from type 2 diabetes, but no assessment for confounding factors was conducted. 

This study has certain strengths. To the best of our knowledge, this prospective, population-based cohort study involved many more participants and incident type 2 diabetes cases than previous studies conducted in a general population. Moreover, this is the first study to investigate the association between human plasma concentrations of BCAAs with incident type 2 diabetes using sensitive measures such as the −2 log likelihood, and testing the robustness of the findings using several sensitivity analyses. 

We are also aware of the limitations of the study. The PREVEND population mainly comprised individuals of European ancestry, which could be translated in an inability to generalize the findings to different ethnicities. We did not have measurements of insulin beyond its baseline assessment, which impedes us in evaluating the evolution of insulin resistance and its association with BCAAs. This fact limits our capacity to describe the biological phenomenon. Finally, because of the absence of repeated BCAA measurements, we are not able to correct for regression dilution, which could have underestimated the BCAA-incident type 2 diabetes associations.

## 5. Conclusions

In conclusion in a population-based cohort, we found that BCAAs were associated with insulin resistance at baseline and with an increased risk of incident type 2 diabetes over 7.5 years of follow-up. Additionally, our results show that BCAA can improve the predictive ability of a conventional risk model. More data are needed to elucidate the interaction between BCAAs and other risk factors during the progression of impaired glucose tolerance.

## Figures and Tables

**Table 1:** Participant characteristics according sex-stratified quartiles of BCAA in participants free of type 2 diabetes at baseline (*n* = 6244).

	All Participants	Quartiles of BCAA				*p*-Value *
		Q1	Q2	Q3	Q4	
		♂ < 365.31	♂ 365.32–408.34	♂ 408.35–454.02	♂ > 454.023	
		♀ < 299.38	♀ 299.39–336.23	♀ 336.24–377.35	♀ > 377.36	
Participants, *n*	6244	1562	1560	1560	1562	
Sex, men, %	49.4	49.4	49.4	49.3	49.4	0.99
Age, y	53.1 ± 11.9	51.76 ± 13.25	52.77 ± 12.31	53.75 ± 12.37	54.34 ± 11.41	<0.0001
Race, white, %	95.4	96.3	96.2	95.8	93.2	<0.0001
Education, high, %	38.0	39.1	41.4	37.8	33.8	<0.001
BMI, kg/m^2^	26.5 ± 4.2	24.7 ± 3.6	25.8 ± 3.7	26.7 ± 3.9	28.6 ± 4.4	<0.0001
SBP, mm Hg	125.6 ± 18.5	123.0 ± 18.6	123.6 ± 17.5	125.8 ± 18.5	130.3 ± 18.6	<0.0001
DBP, mm Hg	73.2 ± 9.0	71.9 ± 9.4	72.5 ± 8.8	73.4 ± 9.0	75.0 ± 8.7	<0.0001
Parental history of CKD, %	0.5	0.6	0.3	0.8	0.3	0.18
Parental history of T2D, %	14.2	12.8	12.5	13.5	18.1	<0.0001
Current smoking status, no %	70.7	65.8	70.8	73.7	72.4	<0.0001
Alcohol intake, never, %	24.1	23.7	22.4	24.5	25.6	0.23
Antihypertensive drugs,%	18.0	14.7	14.5	18.2	24.5	<0.0001
Lipid-lowering drugs, %	7.0	5.1	5.5	7.5	9.8	<0.0001
Total BCAA, μM	370.34 ± 88.63	<365.31	365.32–408.34	408.35–454.02	>454.023	
Valine, μM	203.08 ± 46.58	156.32 ± 50.40	194.59 ± 20.71	215.14 ± 21.63	246.30 ± 31.10	<0.0001
Leucine, μM	124.68 ± 32.56	92.85 ± 31.17	118.95 ± 16.50	132.01 ± 18.48	154.90 ± 25.31	<0.0001
Isoleucine, μM	42.90 ± 16.13	30.01 ± 12.73	39.45 ± 10.35	45.02 ± 11.37	56.67 ± 16.43	<0.0001
TC, mmol/L	5.43 ± 1.03	5.22 ± 0.95	5.36 ± 1.02	5.51 ± 1.04	5.67 ± 1.06	<0.0001
HDL-C, mmol/L	1.25 ± 0.32	1.131 ± 0.37	1.129 ± 0.30	1.25 ± 0.29	1.17 ± 0.28	<0.0001
TG, mmol/L	1.09 (0.79–1.57)	0.88 (0.65–1.20)	1.01 (0.75–1.38)	1.13 (0.84–1.61)	1.47 (1.06–2.08)	<0.0001
Glucose, mmol/L	4.84 ± 0.64	4.872 ± 0.60	4.75 ± 0.59	4.85 ± 0.60	5.03 ± 0.70	<0.0001
Insulin, mU/L	8.00 (5.70–11.80)	6.50 (4.80–8.72)	7.20 (5.20–10.25)	8.30 (6.00–11.70)	11.845 (7.70–16.62)	<0.0001
Serum creatinine, µmol/L	84.55 ± 20.64	83.43 ± 30.66	83.97 ± 16.32	85.14 ± 15.35	85.64 ± 16.16	0.132
eGFR, mL/min/1.73m^2^	92.80 ± 17.00	95.45 ± 17.64	93.57 ± 16.51	91.79 ± 16.33	90.38 ± 17.05	<0.0001
UAE, mg/24h	8.53 (6.02–15.08)	8.15 (5.94–13.82)	8.23 (5.88–13.62)	8.41 (5.98–14.85)	9.65 (6.50–18.20)	0.101
HOMA-IR, (mU mmol/L^2^)/22.5	1.70 (1.17–2.61)	1.40 (1.02–1.93)	1.54 (1.10–2.24)	1.78 (1.25–2.60)	2.53 (1.66–3.85)	<0.0001
HOMA-β, %	132.5 (90.6–200.0)	116.9 (82.5–178.6)	128.5 (90.0–190.0)	133.3 (96.0–193.5)	160.0 (110.5–237.1)	<0.0001
HOMA-β/HOMA-IR	79.78 (50.90–113.63)	79.79 (55.1–113.6)	79.79 (55.1–113.6)	79.79 (50.9–113.6)	60.0 (40.9–88.9)	<0.0001

Continuous variables are reported as mean ± SD, median (interquartile range) and categorical variables are reported as percentage. * Determined by linear-by-linear association chi-square test (categorical variables) and linear regression (continuous variables). Abbreviations: BCAAs, branched-chain amino acids; BMI, body mass index; SBP, systolic blood pressure; DBP, diastolic blood pressure; CKD, chronic kidney disease; T2D, type 2 diabetes; TC, total cholesterol; HDL-C, high-density lipoprotein cholesterol; TG, triglycerides; eGFR, estimated glomerular filtration rate; UAE, urinary albumin excretion; HOMA, Homeostasis Model Assessment; IR, Insulin Resistance.

**Table 2:** Cross-sectional associations of BCAAs with insulin resistance and pancreatic β-cell function.

	HOMA-IR, (mU mmol/L^2^)/22.5		HOMA-β, %	
	β (95% CI)	*p*-Value	β (95% CI)	*p*-Value
Crude Model	28.92 (27.16, 30.67)	<0.0001	20.46 (18.67, 22.25)	<0.0001
Model 1	26.80 (25.18, 28.43)	<0.0001	21.01 (19.38, 22.64)	<0.0001
Model 2	30.95 (27.83, 34.07)	<0.0001	−4.73 (−7.77, −1.70)	0.002

Unstandardized regression coefficients are shown. Model 1: Adjustment for age and sex. Model 2: Model 1 + HOMA-β (for HOMA-IR) and HOMA-IR (for HOMA-β).

**Table 3:** Uni- and multivariable linear regression analyses with BCAA as dependent variable.

Variables	Univariable		Multivariable	
	β (95% CI)	*p*-Value	β (95% CI)	*p*-Value
Sex, female vs. male	69.29 (65.24, 73.33)	<0.0001	55.31 (48.32, 62.30)	<0.0001
Age, years/10	0.58 (0.39, 0.76)	<0.0001	−0.04 (−0.38, 0.31)	0.828
Caucasian, yes vs. no	7.61 (2.36, 12.86)	0.0004	5.73 (0.01, 11.44)	0.049
BMI, kg/m^2^	5.17 (4.67, 5.68)	<0.0001	2.44 (1.76, 3.13)	<0.0001
High education, yes vs. no	0.20 (−0.87, 1.28)	0.7107	0.40 (−0.67, 1.48)	0.463
SBP, mm Hg	0.81 (0.70, 0.93)	<0.0001	−0.09 (−0.31, 0.12)	0.893
DBP, mm Hg	1.92 (1.69, 2.16)	<0.0001	0.15 (−0.27, 0.57)	0.392
Parental history of CKD, yes vs. no	9.50 (−20.38, 39.38)	0.533	8.44 (−24.63, 41.52)	0.616
Parental history of T2D, yes vs. no	10.22 (3.94, 16.51)	0.0001	7.91 (1.03, 14.80)	0.024
Current smoking, yes vs. no	−7.94 (−12.85, −3.03)	0.0001	−4.76 (−10.52, 1.01)	0.105
Alcohol consumption, yes vs. no	11.51 (6.36, 16.66)	<0.0001	9.01 (3.06, 14.96)	0.003
Antihypertensive drugs, yes vs. no	24.35 (18.55, 30.14)	<0.0001	3.78 (−3.19, 10.75)	0.287
Lipid-lowering drugs, yes vs. no	24.87 (16.22, 33.52)	<0.0001	8.16 (−1.48, 17.80)	0.097
TC, mmol/L	7.04 (4.92, 9.15)	<0.0001	4.38 (1.58, 7.19)	0.002
HDL-C, mmol/L	−60.34(−67.05, −53.63)	<0.0001	−20.54 (−30.64,−10.44)	<0.0001
TG, mmol/L	25.51 (23.23, 27.78)	<0.0001	3.61 (0.61, 6.62)	0.018
Serum creatinine, µmol/L	71.16 (61.64, 80.68)	<0.0001	12.23 (−6.78, 31.24)	0.207
eGFR, mL/min/1.73m^2^	−0.44 (−0.57, −0.31)	<0.0001	−0.01 (−0.29, 0.27)	0.956
UAE, mg/24h	0.01 (−0.00, 0.03)	0.095	−0.02 (−0.04, −0.01)	0.005
HOMA-IR, (mU mmol/L^2^)/22.5	28.92 (27.16, 30.67)	<0.0001	22.21 (17.82, 26.59)	<0.0001
HOMA-β, %	20.46 (18.67, 22.25)	<0.0001	−0.89 (−5.01, 3.24)	0.673

Unstandardized regression coefficients are shown. Abbreviations: BCAAs, branched-chain amino acids; BMI, body mass index; SBP, systolic blood pressure; DBP, diastolic blood pressure; CKD, chronic kidney disease; T2D, type 2 diabetes; TC, total cholesterol; HDL-C, high density lipoprotein cholesterol; TG, triglycerides; eGFR, estimated glomerular filtration rate; UAE, Urinary albumin excretion; HOMA, Homeostasis Model Assessment; IR, Insulin Resistance.

**Table 4:** Prospective associations of BCAA with risk of Type 2 Diabetes.

	Q1	Q2	*p*-Value	Q3	*p*-Value	Q4	*p*-Value	BCAA Per 1 SD Increment	*p*-Value
Participants, *n*	1561	1561		1561		1561		6244	
Events, *n*	27	44		72		158		301	
		HR (95% CI)		HR (95% CI)		HR (95% CI)		HR (95% CI)	
Crude Model	(ref)	1.65 (1.01, 2.66)	0.042	2.67 (1.71, 4.14)	<0.0001	6.15 (4.08, 9.24)	<0.0001	1.80 (1.64, 1.98)	<0.0001
Model 1	(ref)	1.58 (0.97, 2.56)	0.064	2.56 (1.62, 4.05)	<0.0001	6.12 (3.92, 9.55)	<0.0001	1.76 (1.59, 1.96)	<0.0001
Model 2	(ref)	1.41 (0.85, 2.32)	0.178	1.87 (1.17, 3.00)	0.009	3.49 (2.19, 5.55)	<0.0001	1.46 (1.29, 1.65)	<0.0001
Model 3	(ref)	1.41 (0.85, 2.33)	0.175	1.87 (1.17, 3.01)	0.008	3.56 (2.24, 5.65)	<0.0001	1.48 (1.31, 1.68)	<0.0001
Model 4	(ref)	1.45 (0.88, 2.41)	0.142	1.84 (1.15, 2.94)	0.010	3.14 (1.99, 4.97)	<0.0001	1.39 (1.23, 1.57)	<0.0001
Model 5a	(ref)	1.50 (0.89, 2.53)	0.124	1.91 (1.17, 3.10)	0.009	2.80 (1.72, 4.53)	<0.0001	1.28 (1.13, 1.46)	0.0001
Model 5b	(ref)	1.59 (0.94, 2.68)	0.079	2.12 (1.30, 3.44)	0.002	3.64 (2.26, 5.87)	<0.0001	1.41 (1.25, 1.60)	<0.0001
Model 5c	(ref)	1.46 (0.87, 2.46)	0.149	1.70 (1.04, 2.77)	0.033	2.32 (1.42, 3.78)	0.0007	1.19 (1.04, 1.35)	0.008

Data are presented as hazard ratios (HRs) with 95% confidence intervals (CIs). Model 1: Model adjusted for age and sex. Model 2: Model 1 + family history of type 2 diabetes and BMI. Model 3: Model 2 + alcohol intake and smoking status. Model 4: Model 3 + TG. Model 5a: Model 4 + HOMA-IR. Model 5b: Model 4 + HOMA-β. Model 5c: Model 4 + HOMA-IR and HOMA-β. Abbreviations: BMI, body mass index; TG, triglycerides; HOMA, Homeostasis Model Assessment; IR, Insulin Resistance.

## Supplementary Materials

The Table S1, Table S2 and Table S3 are available online at http://www.mdpi.com/2077-0383/7/12/513/s1.
