# Supplementary material for: Plasma Branched-Chain Amino Acids and Risk of Incident Type 2 Diabetes: Results from the PREVEND Prospective Cohort Study
Source: J Clin Med. 2018 Dec 4;7(12):513. doi: 10.3390/jcm7120513 (PMC6306832; doi:10.3390/jcm7120513)
Supplement: Supplementary file 1 [file jcm-07-00513-s001.pdf]

Table S1. Prospective associations of Valine with risk of Type 2 Diabetes.

	Q1	Q2	*p*-Value	Q3	*p*-Value	Q4	*p*-Value	Valine Per 1 SD Increment	*p*-Value
	≤179.80	179.81–204.17		204.18–230.02		>230.02			
Participants, *n*	1561	1561		1561		1561		6244	
Events, *n*	30	46		70		155		301	
		HR (95% CI)		HR (95 % CI)		HR (95% CI)		HR (95% CI)	
Crude Model	(ref)	1.52 (0.96, 2.42)	0.072	2.31 (1.50, 3.54)	0.0001	5.34 (3.61, 7.90)	<0.0001	1.71 (1.55, 1.88)	<0.0001
Model 1	(ref)	1.39 (0.87, 2.22)	0.161	2.06 (1.33, 3.21)	0.0001	4.68 (3.07, 7.14)	<0.0001	1.63 (1.46, 1.88)	<0.0001
Model 2	(ref)	0.98 (0.60, 1.60)	0.958	1.40 (0.89, 2.21)	0.137	2.42 (1.56, 3.75)	<0.0001	1.33 (1.17, 1.51)	<0.0001
Model 3	(ref)	0.99 (0.61, 1.61)	0.975	1.42 (0.90, 2.23)	0.126	2.48 (1.60, 3.84)	<0.0001	1.35 (1.19, 1.54)	<0.0001
Model 4	(ref)	1.01 (0.62, 1.64)	0.957	1.42 (0.90, 2.23)	0.126	2.35 (1.52, 3.63)	0.0001	1.34 (1.18, 1.51)	<0.0001
Model 5a	(ref)	1.14 (0.69, 1.91)	0.592	1.46 (0.90, 2.35)	0.120	1.88 (1.17, 3.01)	0.008	1.16 (1.01, 1.32)	0.026
Model 5b	(ref)	1.11 (0.66, 1.87)	0.672	1.62 (1.00, 2.63)	0.047	2.71 (1.70, 4.31)	<0.0001	1.40 (1.23, 1.59)	<0.0001
Model 5c	(ref)	1.08 (0.64, 1.80)	0.757	1.41 (0.87, 2.28)	0.158	1.68 (1.04, 2.71)	0.033	1.13 (0.98, 1.29)	0.07

Data are presented as hazard ratios (HR) with 95 % confidence intervals (CI). Model 1: Model adjusted for Age and Sex. Model 2: Model 1 + family history of type 2 diabetes and BMI. Model 3: Model 2 + alcohol intake and smoking status. Model 4: Model 3 + TG. Model 5a: Model 4 + HOMA-IR. Model 5b: Model 4 + HOMA-β. Model 5c: Model 4 + HOMA-IR and HOMA-β. Abbreviations: BMI, body mass index; TG, triglycerides; HOMA, Homeostasis Model Assessment; IR, Insulin Resistance.

Table S2. Prospective associations of Leucine with risk of Type 2 Diabetes.

	Q1	Q2	*p*-Value	Q3	*p*-Value	Q4	*p*-Value	Leucine Per 1 SD Increment	*p*-Value
	≤106.36	106.37–124.31		124.32–143.32		>143.32			
Participants, *n*	1561	1561		1561		1561		6244	
Events, *n*	29	48		72		152		301	
		HR (95% CI)		HR (95 % CI)		HR (95 % CI)		HR (95% CI)	
Crude Model	(ref)	1.63 (1.02, 2.59)	0.038	2.47 (1.60, 3.80)	<0.0001	5.40 (3.63, 8.04)	<0.0001	1.80 (1.62, 2.01)	<0.0001
Model 1	(ref)	1.55 (0.97, 2.47)	0.062	2.28 (1.46, 3.55)	0.0002	4.97 (3.25, 7.55)	<0.0001	1.74 (1.54, 1.95)	<0.0001
Model 2	(ref)	1.29 (0.80, 2.09)	0.284	1.68 (1.07, 2.64)	0.023	3.05 (1.98, 4.71)	<0.0001	1.47 (1.30, 1.67)	<0.0001
Model 3	(ref)	1.31 (0.81, 2.11)	0.261	1.72 (1.09, 2.69)	0.018	3.07 (1.99, 4.74)	<0.0001	1.48 (1.30, 1.68)	<0.0001
Model 4	(ref)	1.32 (0.82, 2.13)	0.248	1.65 (1.05, 2.59)	0.027	2.69 (1.75, 4.13)	<0.0001	1.35 (1.20, 1.53)	<0.0001
Model 5a	(ref)	1.46 (0.88, 2.42)	0.136	1.67 (1.04, 2.69)	0.033	2.14 (1.34, 3.42)	0.001	1.21 (1.07, 1.38)	0.002
Model 5b	(ref)	1.44 (0.87, 2.42)	0.152	1.93 (1.19, 3.14)	0.006	2.99 (1.88, 4.73)	<0.0001	1.39 (1.23, 1.58)	<0.0001
Model 5c	(ref)	1.35 (0.81, 2.23)	0.242	1.66 (1.03, 2.67)	0.036	1.90 (1.18, 3.05)	0.007	1.18 (1.03, 1.34)	0.011

Data are presented as hazard ratios (HR) with 95 % confidence intervals (CI). Model 1: Model adjusted for Age and Sex. Model 2: Model 1 + family history of type 2 diabetes and BMI. Model 3: Model 2 + alcohol intake and smoking status. Model 4: Model 3 + TG. Model 5a: Model 4 + HOMA-IR. Model 5b: Model 4 + HOMA-β. Model 5c: Model 4 + HOMA-IR and HOMA-β. Abbreviations: BMI, body mass index; TG, triglycerides; HOMA, Homeostasis Model Assessment; IR, Insulin Resistance.

Table S3. Prospective associations of Isoleucine with risk of Type 2 Diabetes.

	Q1	Q2	*p*-Value	Q3	*p*-Value	Q4	*p*-Value	Isoleucine Per 1 SD Increment	*p*-Value
	≤32.54	32.55–41.98		41.99–52.00		>52.01			
Participants, *n*	1543	1543		1543		1543		6172	
Events, *n*	38	40		84		137		299	
		HR (95% CI)		HR (95 % CI)		HR (95 % CI)		HR (95% CI)	
Crude Model	(ref)	1.02 (0.65, 1.59)	0.928	2.23 (1.52, 3.27)	<0.0001	3.71 (2.60, 5.34)	<0.0001	1.64 (1.50, 1.78)	<0.0001
Model 1	(ref)	1.01 (0.68, 1.59)	0.945	2.16 (1.45, 3.21)	0.0001	3.70 (2.49, 5.48)	<0.0001	1.64 (1.49, 1.81)	<0.0001
Model 2	(ref)	0.81 (0.51, 1.30)	0.397	1.68 (1.12, 2.52)	0.012	2.29 (1.53, 3.44)	<0.0001	1.39 (1.25, 1.54)	<0.0001
Model 3	(ref)	0.81 (0.51, 1.29)	0.387	1.69 (1.13, 2.54)	0.010	2.34 (1.56, 3.51)	<0.0001	1.41 (1.27, 1.56)	<0.0001
Model 4	(ref)	0.85 (0.53, 1.36)	0.517	1.73 (1.15, 2.60)	0.007	2.09 (1.40, 3.11)	0.0002	1.27 (1.15, 1.41)	<0.0001
Model 5a	(ref)	0.85 (0.52, 1.38)	0.522	1.45 (0.94, 2.22)	0.085	1.57 (1.02, 2.40)	0.037	1.14 (1.02, 1.27)	0.017
Model 5b	(ref)	0.84 (0.52, 1.38)	0.513	1.83 (1.20, 2.81)	0.004	2.43 (1.59, 3.69)	<0.0001	1.32 (1.19, 1.47)	<0.0001
Model 5c	(ref)	0.81 (0.50, 1.32)	0.411	1.42 (0.93, 2.18)	0.103	1.59 (1.03, 2.45)	0.033	1.11 (1.00, 1.24)	0.043

Data are presented as hazard ratios (HR) with 95 % confidence intervals (CI). Model 1: Model adjusted for Age and Sex. Model 2: Model 1 + family history of type 2 diabetes and BMI. Model 3: Model 2 + alcohol intake and smoking status. Model 4: Model 3 + TG. Model 5a: Model 4 + HOMA-IR. Model 5b: Model 4 + HOMA-β. Model 5c: Model 4 + HOMA-IR and HOMA-β. Abbreviations: BMI, body mass index; TG, triglycerides; HOMA, Homeostasis Model Assessment; IR, Insulin Resistance.
